# Chimeric Antigen Receptor (CAR) T Cell Therapy for Metastatic Melanoma: Challenges and Road Ahead

**DOI:** 10.3390/cells10061450

**Published:** 2021-06-09

**Authors:** Tahereh Soltantoyeh, Behnia Akbari, Amirali Karimi, Ghanbar Mahmoodi Chalbatani, Navid Ghahri-Saremi, Jamshid Hadjati, Michael R. Hamblin, Hamid Reza Mirzaei

**Affiliations:** 1 Department of Medical Immunology, School of Medicine, Tehran University of Medical Sciences, Tehran 1417613151, Iran; tahereh.soltantoye@gmail.com (T.S.); behniaceae@gmail.com (B.A.); ghanbarmahmoodi@gmail.com (G.M.C.); navidnt75@gmail.com (N.G.-S.); hajatij@tums.ac.ir (J.H.); 2 School of Medicine, Tehran University of Medical Sciences, Tehran 1417613151, Iran; karimi.amirali.1999@gmail.com; 3 Laser Research Centre, Faculty of Health Science, University of Johannesburg, Doornfontein 2028, South Africa; hamblin@helix.mgh.harvard.edu; 4 Radiation Biology Research Center, Iran University of Medical Sciences, Tehran 1449614535, Iran

**Keywords:** metastatic melanoma, chimeric antigen receptor T cells, immunotherapy

## Abstract

Metastatic melanoma is the most aggressive and difficult to treat type of skin cancer, with a survival rate of less than 10%. Metastatic melanoma has conventionally been considered very difficult to treat; however, recent progress in understanding the cellular and molecular mechanisms involved in the tumorigenesis, metastasis and immune escape have led to the introduction of new therapies. These include targeted molecular therapy and novel immune-based approaches such as immune checkpoint blockade (ICB), tumor-infiltrating lymphocytes (TILs), and genetically engineered T-lymphocytes such as chimeric antigen receptor (CAR) T cells. Among these, CAR T cell therapy has recently made promising strides towards the treatment of advanced hematological and solid cancers. Although CAR T cell therapy might offer new hope for melanoma patients, it is not without its shortcomings, which include off-target toxicity, and the emergence of resistance to therapy (e.g., due to antigen loss), leading to eventual relapse. The present review will not only describe the basic steps of melanoma metastasis, but also discuss how CAR T cells could treat metastatic melanoma. We will outline specific strategies including combination approaches that could be used to overcome some limitations of CAR T cell therapy for metastatic melanoma.

## 1. Introduction

Melanoma is the most lethal type of skin cancer [1,2,3], which develops from uncontrolled proliferation of the melanin producing cells within the skin, called melanocytes [1,4]. Patients with melanoma are staged based on the 2009 American Joint Committee on Cancer (AJCC) staging system [5]. Based on location of the primary tumor, tumor size, number of tumors, lymph node involvement, and the presence or absence of metastasis, melanoma is classified into four stages [6]. According to the melanoma staging systems, patients with stage IV melanoma show tumor spread throughout the body, called metastatic melanoma [6,7,8,9]. Strategies for treatment of metastatic melanoma include: chemotherapy, radiation, targeted therapy, and immunotherapy [6,10,11]. Because of their non-specificity and treatment resistance, chemotherapy and radiation therapy are not considered to be good options for the treatment of metastatic melanoma [6]. New pharmaceutical agents, including anti-PD-1 checkpoint blockade immunotherapy, and B-RAF inhibitor targeted therapy have both been approved for metastatic melanoma. However, B-RAF inhibitors lead to treatment resistance, while checkpoint blockades can cause autoimmune disease [12,13,14]. Thus, researchers are still seeking new treatments for metastatic melanoma. Recently, adoptive T cell therapy (ACT) has been investigated as a new strategy for improving the treatment of metastatic melanoma [11]. CAR T cells kill tumor cells through recognition of target antigens on the surface of tumor cells in a non-MHC restricted manner. Upon antigen recognition, CAR T cells release various cytotoxic molecules such as granzyme and perforin as well as cytokines, leading to tumor cell apoptosis and lysis [15]. CAR-T cells are genetically modified T lymphocytes that express chimeric antigen receptors, with three main regions: extracellular, transmembrane, and intracellular domains [16]. The extracellular domain contains the scFv (single chain variable fragment) of an antibody that targets a tumor antigen in a non-MHC restricted manner. The scFv domain is linked to the intracellular domain CD3z with or without additional co-stimulatory domains in the intracellular region by the transmembrane region to trigger T lymphocyte activation [16]. Based on the number of intracellular domains and inclusion of additional genes to CAR transgene, CAR T cells have been classified into five generations. First generation of CAR consists of CD3ζ molecules. Second generation CAR includes CD3ζ and one co-stimulatory molecule (e.g., CD28 or 4-1BB) while third generation CAR consists of CD3ζ and two co-stimulatory molecules (e.g., CD28 and 4-1BB or OX-40 and CD28). The fourth generation of CAR is designed based on second generation CAR paired with an inducible or constitutively expressed cytokine or chemokine (e.g., IL-12). The fifth generation of CAR is also based on second generation CARs coupled with truncated cytoplasmic domain of particular cytokine receptors with a binding site for specific transcription factors such as STAT3/5. [17,18]. CAR T cell therapy has been shown to be a potent immunotherapeutic approach for the treatment of hematologic malignancies, and two types of CAR T cells have been approved by the US Food and Drug Administration (FDA) for the treatment of B-cell malignancies [19]. Nevertheless, there remain challenges, such as selecting the proper target antigen, the immunosuppressive tumor microenvironment (TME), and barriers preventing the infiltration of CAR T cells into the tumor microenvironment, which lower the efficacy against solid tumors [20]. In this present paper, we review the mechanisms of melanoma metastasis to find suitable antigen targets, summarize the preclinical and clinical studies of CAR T cell therapy in metastatic melanoma, together with the advantages and disadvantages, and provide some suggestions to increase treatment efficiency against metastatic melanoma.

## 2. Mechanisms of Melanoma Metastasis: Implications for CAR T Cell Therapy

**Figure 1:**
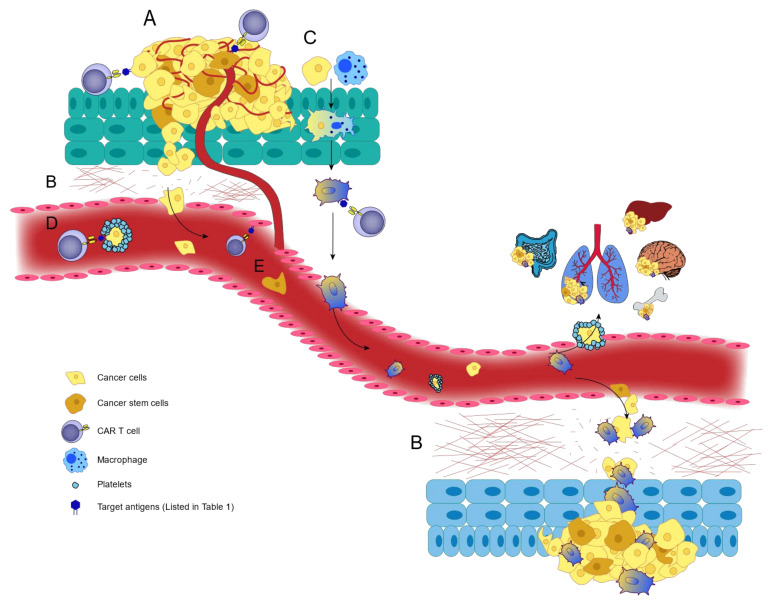
Mechanisms of melanoma metastasis. The melanoma metastasis process can take place via lymphatic route or blood vessels by various means. (**A**) Angiogenesis. Several agents including VEGFs, growth factors, integrins contribute to angiogenesis by binding to their receptors on endothelial cells. (**B**) Intravasation, extravasation and matrix degradation. Melanoma cells migrate to distant organs, requiring adhesion molecules and MMPs to intravasate into and extravasate out of vessels, and degrade the ECM to cause metastasis. (**C**) Leukocyte-cancer cell fusion hypothesis. Melanoma cells can fuse with leukocytes, especially macrophages, and cause metastasis following epigenetic reprograming. (**D**) Embolisms. Platelet aggregation facilitates melanoma cell metastasis by protecting them from NK cell cytolytic activity and increasing their migration by platelet-tumor cell interactions. (**E**) Cancer stem cells. These cells have the potential to induce angiogenesis, matrix degradation, intravasation, and extravasation to promote melanoma metastasis.

Although regional lymph node involvement (stage III) is a part of the metastasis process, according to the staging system, only stage IV in which tumor cells metastasize to distant organs is considered as metastatic melanoma [21]. Melanoma cells mostly metastasize through the lymphatic route [21], but the hematogenous route also seems to be involved in some cases [22]. Herein, we will not only review the mechanisms of metastasis that lead to lymph node involvement, hematogenous spread, and the involvement of distant organs (Figure 1), but also how selection of an ideal antigen which has a high surface expression level on cancerous tissues and low surface expression level on normal tissues is critical and could significantly decrease the risk of CAR T cell mediated off-tumor toxicity [23]. However, the complete mechanisms of melanoma metastasis are not yet fully understood [21].

### 2.1. Angiogenesis

Angiogenesis describes the process by which new blood vessels are formed from pre-existing blood vessels, and is one of the most important factors involved in tumor progression and metastasis [24,25]. Several agents in the TME can stimulate angiogenesis through different mechanisms [21]. One of the most important causes of angiogenesis is the family of vascular endothelial growth factors (VEGFs) [26]. VEGF-A as a member of the VEGFs family is largely linked to angiogenesis [27], and its expression level is up-regulated in melanoma cells in a hypoxia condition [27]. Data has been shown that VEGF-A may be involved in the progression of metastasis [28]. VEGF-A alone or VEGF-A/PGF heterodimer contributes to VEGFR2 activation [28,29]. In line with this finding, studies have pointed out that VEGFR2 activation predominantly mediates angiogenesis response [29]. In addition to VEGFs, other growth factors, cytokines, and integrins, including basic fibroblast growth factor (bFGF), placental growth factor (PGF), urokinase plasminogen activator [30], IL8, αvβ3 and αvβ5 integrins, and angiopoietins play important roles in melanoma angiogenesis [25]. Melanoma cells secrete other growth factors, including bFGF (FGF2) and PGFs. bFGF is a potent angiogenic factor that regulates many cellular functions such as angiogenesis [31]. It has been shown that the action of matrix metalloproteinase results in bFGF release, which binds to its receptor on endothelial cells, FGFR1. This interaction promotes melanoma metastasis by regulating endothelial cell proliferation and increasing angiogenesis [32,33].

PGFs (PGF1 and PGF2) interact with VEGFR1 and also with neuropilin-1 and neuropilin-2 receptors on endothelial cells. PGFs enhances angiogenesis in two ways: direct effects on pre-existing endothelial cells, and indirect effects, by recruitment of VEGFR-1 positive hematopoietic precursor cells from the bone marrow to blood vessels [34,35]. 

Urokinase plasminogen activator, which binds to the uPAR receptor, plays a role in melanoma progression and metastasis [36]. uPAR is expressed on both endothelial and melanoma tumor cells [25]. uPAR regulates angiogenesis by increasing endothelial cell migration and organization into tube-like structures [37].

IL-8 is another molecule with a significant role in angiogenesis. IL-8 binds to G-protein-coupled receptors (GPCRs), including CXCR1 and CXCR2 on endothelial cells, and induces endothelial cell migration and permeability. Experimental studies have shown that IL-8 over-expression could increase angiogenesis and melanoma metastasis [38,39]. 

Studies have also shown that lymphangiogenesis plays an important role in the spread of melanoma to regional lymph nodes, and in metastasis [40].

VEGF-C secreted from melanoma cells can bind to VEGFR2 and VEGFR3 on lymphatic endothelial cells [41] and promote formation of lymphatic vessels and increase lymph node metastasis [42,43]. In 2008, Sini and colleagues have reported that inhibition of VEGFRs reduced lymph node metastasis in an animal model of metastatic melanoma [44].

In addition to cellular adhesion, invasion and migration, αvβ3 and αvβ5 integrins expressed on endothelial cells can regulate angiogenesis by modulating VEGF and bFGF [30,45]. These pathways play a crucial role in the progression of localized melanoma to metastatic melanoma [46].

### 2.2. Extravasation and Intravasation

Intravasation is the process by which a single or a group of melanoma cells, which have become detached from the primary tumor, can enter the blood or lymphatic vasculature system [47]. After intravasation, tumor cells move out of the vessels into the surrounding tissues (extravasation) [48]. In these steps, the melanoma cells require adhesion molecules to stick to the endothelial cells, and proteolytic enzymes to invade into the extracellular matrix (ECM) [21,47]. 

Adhesion molecules include cadherins, integrins, and the immunoglobulin superfamily. Matrix metalloproteinases (MMPs) also play an important role in intravasation and extravasation, which are two important steps in melanoma metastasis [47].

#### 2.2.1. Cadherins

Melanoma cells undergo loss of expression of E (epithelial)-cadherin, and at the same time gain expression of N (neural) cadherin. N-cadherin is a classical cadherin which leads to adhesion of melanoma cells to each other, and to other N-cadherin expressing cells such as endothelial cells [47]. An in vivo study in immunocompromised mice showed that silencing of N-cadherin inhibited melanoma cell extravasation and lung metastasis [49]. Therefore, this membrane protein may be a potential target for CAR T cell therapy for metastatic melanoma.

#### 2.2.2. Integrins

Integrins are a family of adhesion molecules that contribute to angiogenesis, tumor cell proliferation, migration and metastasis, by cell–cell or cell–matrix interactions [50]. Expression of integrin αvβ3 on melanoma cells led to increased metastasis to the lungs [51]. Melanoma cells that express integrin α4β1 tend towards lymph node metastasis [52] through binding to vascular cell adhesion molecule-1 (VCAM-1) on endothelial cells [53]. Integrin α4β1 also facilitated migration of CCR9 bearing melanocytes to the small intestine [54]. As melanoma cells do not express B2-integrins (LFA-1 or Mac-1) on their surface, these cells can bind to the B2-integrins of neutrophils by their intercellular adhesion molecule-1 (ICAM1), and then move into the vessels [48]. During angiogenesis, the endothelial cells over-express αv-integrins that can bind to lactadherin (also known as milk fat globule-EGF factor 8 protein) on melanoma cells, thus increasing adhesion and migration [55]. The α6β4 integrin on melanoma tumor cells is able to interact with lung endothelial cell adhesion molecule-1 (Lu-ECAM or CLCA2), that is expressed on lung cells and can lead to lung metastasis [56]. Because of the role of integrins in angiogenesis, tumor growth, and metastasis, several integrin inhibitors are under investigation in clinical trials [57]. Most of the clinical trials are studying αvβ3 integrin inhibitors [58]. 

#### 2.2.3. Immunoglobulin Superfamily (IgSF)

Several members of the IgSF, including MCAM (CD146), NCAM (CD56), ALCAM (CD166), and L1-CAM (CD171) have been associated with metastasis in several cancers such as melanoma [59]. The melanoma cell adhesion molecule (MCAM/MUC18), also known as CD146, is a member of the immunoglobulin superfamily, and a cell adhesion molecule that mediates adhesion between melanoma cells themselves, as well as adhesion between melanoma cells and endothelial cells [60]. An in vivo study showed that an anti-MCAM/MUC18 antibody inhibited melanoma growth and metastasis [61]. Activated leukocyte cell adhesion molecule (ALCAM), CD166, or MEMD is a type I membrane protein and another member of the IgSF. Over-expression of ALCAM increased melanoma cell aggregation and metastasis [62,63,64]. Studies have shown that blocking of ALCAM by the secreted variant of ALCAM diminished metastatic capacity in nude mice [65]. The interaction between NCAM and CD56, that mediates cell–cell adhesion, is expressed on several tumor types such as melanoma, where it increases metastasis [66]. An in vivo study demonstrated that the silencing of NCAM expression inhibited melanoma cell invasion and metastasis [67]. L1-CAM is another cell adhesion molecule, and its over-expression was associated with melanoma metastasis [68]. L1-CAM knock-down reduced metastasis in a melanoma xenograft model [69]. Platelet endothelial cell adhesion molecule 1 (PECAM-1) or CD31 is another member of the IgSF that is expressed on endothelial cells. Melanoma cells which over-express heparan sulfates can interact with endothelial cells by binding to PECAM-1 [70]. It was shown that the heparan sulfate-PECAM-1 interaction contributed to tumor cell arrest and extravasation [59]. An in vivo study showed that an anti-PECAM-1 mAb inhibited metastasis in melanoma [71], and could be a potential target for CAR T cell therapy in metastatic melanoma.

Platelet factor 4 (PF4) or CXCL4 is a protein that can inhibit tumor metastasis by decreasing blood vessel integrity, angiogenesis inhibition, increasing myeloid-derived suppressor cells (MDSCs), and hematopoietic stem cells (HSCs) [72,73]. As such, based on studies [74], the PF4/CXCL4 recombinant protein could be a therapeutic option in combination with CAR T cell therapy for metastatic melanoma by preventing angiogenesis. 

#### 2.2.4. Matrix Metalloproteinases

MMPs are endopeptidase enzymes [75] that degrade and remodel the extracellular matrix (ECM) via proteolytic activity, and are involved in invasion and metastasis [76]. Members of this family include both soluble and membrane-bound types [77]. Although the soluble forms of MMPs may not be a direct target for CAR T cell therapy, they can be used indirectly in combination with CAR T cell therapy against other targets. Thus in this section, we will cover both soluble and membrane bound MMPs. Over-expression of MMP-1, 2, 9, 13 and 14 was shown to occur in invasive melanoma [76]. Studies have shown that inhibition of MMP-1 decreases melanoma metastasis in nude mice [78], whereas forced over-expression of MMP-1 in non-invasive melanoma cells induced a metastatic phenotype [79]. Moreover, the role of MMP-2 in human melanoma invasion and metastasis is also important [80]. Membrane type-1 MMP (MT1-MMP) (or MMP-14) is a cell–surface membrane protein, which activates MMP-2 leading to matrix degradation and melanoma cell invasion and metastasis [81]. MT1-MMP can also degrade ECM directly by cleavage of ECM components, such as collagens, laminin, and fibrins [77]. The expression of MT1-MMP on endothelial cells contributes to angiogenesis by ECM remodeling and promotion of vessel growth [82]. Active MMP-2 can either be membrane-bound or secreted [76]. The secreted form can bind to integrin αvβ3 and facilitate matrix degradation and melanoma cell migration [83]. The cell-surface hyaluronan receptor, CD44, binds to MMP9 on melanoma cells and forms a CD44/MMP9 complex. It has been shown that disruption of the CD44/MMP9 complex inhibits tumor invasion [84]. Based on an in vivo study, stromal derived MMP-13 (a collagenolytic enzyme) is also required for melanoma metastasis [85]. However, MMP-8 (a collagenase II enzyme) has anti-tumor and anti-metastatic activity in cancers such as melanoma. Therefore, CAR T cells that additionally express MMP-8 may be a good combination therapy for treatment of metastatic melanoma [86]. 

### 2.3. Leukocyte-Cancer Cell Fusion Hypothesis

In 1992, John Pawelek, discovered metastatic melanoma cells that resembled fused macrophage-melanoma hybrid cells [87]. In vitro studies have shown that melanoma-macrophage hybrids showed markedly higher tumorigenicity and metastatic capacity [88,89]. Macrophage and cancer cell fusion results in epigenetic reprogramming. The metastatic hybrid cells increase the expression of macrophage markers, including SPARC, SNAIL, MET, MITF, CD14, CD68, CD163, CD204 and CD206. They also express integrin subunits including α3, α5, α6, αv, β1, β3; GnT-V (β1,6-acetylglucosaminyltransferase-V) and its enzymatic products, β1,6-branched oligosaccharides and cell-surface LAMP1. These molecules lead to increased tumorigenicity and metastatic potential in these melanoma hybrid cells [90,91]. The hybrid cells also express melanocyte markers (ALCAM, MLANA), and stem cell markers (CD44, CXCR4) [91]. The glycan molecules, β1,6-branched oligosaccharides, contribute to motility, invasion and metastasis of melanoma by influencing the adhesion to extracellular matrix components [92,93]. Secreted protein acidic and rich in cysteine (SPARC) or osteonectin is a extracellular protein [94] that increases tumor metastasis by driving vascular permeability and extravasation [95]. Snail is a transcription factor that induces epithelial-mesenchymal transition (EMT- EMT is a crucial characteristic of invading cancer cells) by suppressing the expression of E-cadherin [96,97]. MET is a receptor tyrosine kinase and membrane-bound protein that binds to hepatocyte growth factor (HGF). In vivo studies have indicated that over-expression of either MET or HGF promotes metastasis [98,99]. Moreover, MET inhibitors have been shown to inhibit metastasis in both melanoma animal models and in patients [100,101,102]. Microphthalmia-associated transcription factor (MITF) is a transcription factor that is required for metastasis in melanoma animal models, and its depletion from melanoma cells decreased lung metastasis [103]. Lysosome-associated membrane protein-1 (LAMP1), also known CD107a, is a surface protein that is expressed on melanoma-macrophage hybrid cells and increases their invasion and metastatic potential [104]. Binding of this molecule to galectin-3 in the lungs is one mechanism that can lead to metastasis [105]. LAMP1 interaction with the ECM in target organs could be another mechanism [106]. Also, LAMP1 is a major carrier of β1,6 branched N-glycans [107]. Down-regulation of LAMP1 significantly reduced the metastatic capacity of melanoma cells [108]. GnT-V is a Golgi membrane-bound protein that catalyzes the formation of 1,6 N-acetylglucosamine in the Golgi apparatus and its down-regulation inhibited metastasis in gastric cancer cells [109]. Therefore, targeting GnT-V, SPARC, SNAIL, MITF in combination with CAR T cells that are specific to surface proteins of MTFs (macrophage-tumor fusion cells), such as β1,6-branched oligosaccharides, MET and LAMP1 may improve CAR T cell performance in metastatic melanoma by eliminating leukocyte-cancer fusion cells. Since CD14 and CD68 are pan-macrophage markers [91], it would be risky to target them, but CD163, CD204 and CD206 are mainly expressed by M2 macrophages, which are the tumor-promoting phenotypes [91]. Therefore, targeting M2 markers may be a good idea for elimination of melanoma-macrophage hybrid cells. MLANA/MART-1 is a melanocyte specific marker that is recognized by T cells. One clinical trial investigated adoptive transfer of MART-1 specific TCR engineered T cells in metastatic melanoma patients. Their results showed potent evidence of tumor regression [110]. Although antibodies against this marker are used for the diagnosis of melanoma [111], this target has not been used for MAb therapy. CD44 is the receptor for hyaluronic acid and a transmembrane glycoprotein that is expressed on cancer stem cells [112]. Among CD44 variants, the CD44v3 splice is associated with metastasis in melanoma patients [113]. The targeting of CD44 variants with MAbs could be a novel immunotherapy for cancer treatment [114], and CD44v3 may be a potential target for CAR T cell therapy for metastatic melanoma. CXCR4 activation on tumor cells is associated with increased metastasis in several cancers by promoting invasion, tumor cell proliferation, matrix degradation, and neoangiogenesis [115]. Interestingly, CXCR4 is widely expressed on both T cells and hematopoietic stem cells [116]. It is possible to design anti-CXCR4 CAR T cells by inserting a CAR construct into the endogenous locus of CXCR4 in T cells using a Crispr/CAS9 approach [117]. However, the use of this molecule as a CAR T target should be further investigated due to its expression on hematopoietic stem cells.

### 2.4. Embolisms

The formation of a tumor cell embolism appears to contribute to hematogenous metastasis. In the emboli, tumor cells form aggregates with leukocytes and platelets [22]. Moreover, prothrombotic agents such as protease-activated thrombin receptor (PAR-1), thrombin, and platelet-specific receptor glycoprotein Ib-IX (gpIb-IX) play a role in embolism formation and melanoma metastasis [118,119]. PAR-1 is a seven transmembrane G-protein-coupled receptor that is expressed on metastatic melanoma cells and activated by proteolytic cleavage of the N-terminal domain of the receptor by serine proteases (especially thrombin) [120]. MMP-1 is also involved in PAR1 activation [121]. Maspin is a tumor suppressor protein that is negatively regulated via PAR-1 in metastatic melanoma. Maspin decreased lung metastasis in melanoma by inhibiting MMP-2 expression and activity [120]. Also, PAR-1 increases expression of connexin-43 in gap junctions, which is critical for tumor cell extravasation in metastatic melanoma [122]. PAR-1 activation increased expression of platelet activating factor receptor (PAFR) and its ligand (PAF). The PAFR/PAF complex can activate platelets and promote tumor-platelet aggregation [123]. Therefore, PAR-1 targeting could be a monotherapy or a combination approach with CAR T cell therapy for treatment of metastatic melanoma [120]. TR47 is a soluble peptide that is generated upon cleavage of PAR-1, and decreased melanoma metastasis in vivo [124]. Therefore, CAR T cells that express TR47 may be a good choice for treatment of metastatic melanoma. Platelet-specific receptor gpIb-IX is a major adhesion protein that is expressed on platelet membranes, and is activated after interaction with different ligands, such as von Willebrand factor (vWF), to form platelet aggregates [125]. Tumor cells express adhesion molecules, such as P-selectin glycoprotein ligand-1 (PSGL-1) and CD44, which bind to P-selectin on activated platelets to form aggregates [126]. Activated platelets in the thrombus protect circulating tumor cells from the cytolytic activity of NK cells by formation of platelet-tumor cell aggregates, and enable melanoma cells to extravasate from the circulation and metastasize to the lungs [119,127]. Although these glycoproteins play a major role in platelet aggregation, it is possible that targeting this receptor may cause platelet disorders including thrombocytopenia.

### 2.5. Cancer Stem Cells

In several solid tumors including metastatic melanoma, cancer stem cells (CSCs) are responsible for resistance to conventional treatment, recurrence, and progression of tumors. In metastatic melanoma CSCs are also known as malignant melanoma stem cells [128]. These cells contribute to melanoma metastasis by promoting neovascularization, angiogenesis, matrix degradation, intravasation, and extravasation. Melanoma stem cells can differentiate into endothelial-like cells and promote neovascularization. They can also lead to angiogenesis by expression of VEGFs. Growth factors and cytokines in the tumor microenvironment can affect melanoma stem cells, and reprogram the expression of transcription factors that are involved in EMT. These cells can degrade the matrix by MMPs, and destroy the endothelial barrier, resulting in intravasation and extravasation. Therefore, melanoma stem cells ultimately promote invasion and metastasis [129]. Melanoma stem cells express specific markers, including CD133, CD20, ABCB5, CD271, and ALDH1 [129]. Studies have shown that targeting of melanoma stem cells using CD133 and CD20 specific monoclonal antibodies attenuated tumor growth and lowered the metastatic potential [130,131]. ABCB5 promotes metastasis by activation of the NF-κB pathway, so this marker could provide a potential therapeutic target [132]. Immunotherapy by administering a CD271 specific antibody effectively suppressed metastasis in a melanoma animal model [133]. Targeting of ALDH1 (aldehyde dehydrogenase) reduced metastasis in melanoma [134]. As such, it appears that these markers may be good targets for CAR T cell therapy. Other candidate markers, including CD166, CXCR4, or neural precursor cell expressed developmentally down-regulated protein 9 (NEDD9), might also be involved in MMSC invasion, migration, and metastasis [128], but further studies are need to confirm their involvement. Other markers such as jumonju AT-rich interactive domain 1B (JARID1B) are expressed on melanoma stem cells [129], but have not been correlated with melanoma invasion or metastasis [135].

### 2.6. Chemotactic Molecules

Chemokines and chemoattractant cytokines bind to their receptors, and play a key role in the metastatic process in several cancers, including melanoma [136]. Takeuchi, in 2004, showed that expression of the CCR7 chemokine receptor on melanoma cells increased the migration of these cells to lymph nodes by binding to the CCL21 chemokine [137]. However, since CCR7 is also expressed on naïve T cells and dendritic cells [138], it is possible that targeting of this receptor, although it could decrease metastasis, could also disrupt the migration of normal immune cells to the lymph nodes and inhibit the anti-tumor response. Therefore, this receptor is not thought to be a specific target for melanoma immunotherapy using CAR T cells. Preclinical studies have shown that CXCR3 expression on melanoma cells increases metastasis to lymph nodes, and that inhibition of CXCR3 by antisense RNA decreased lymph node metastasis [139]. IGF-1R and CXCR4 showed higher expression on uveal melanoma cells compared to normal melanocytes. Therefore, because of the high expression of the corresponding ligands (IGF and CXCL12, respectively) in the liver, uveal melanoma most often shows liver metastasis [140,141]. Moreover, CCL25 which is produced by small intestinal epithelial cells recruits CCR9-expressing melanoma cells [54]. 

In conclusion, several MAbs and antagonists have been used to block chemokine receptors in melanoma to inhibit metastasis. Application of CAR T cells to target chemokines and receptors must be carefully investigated and monitored due to their wide expression, and their role in recruitment of immune cells to tumor sites. It is possible that SynNotch receptor CAR T cells or tandem CAR T cells could reduce the off-target toxicity of CAR T cells that recognize chemotactic molecules. Further, using the fourth generation TRUCK (T cells redirected for antigen-unrestricted cytokine-initiated killing) CAR T cells should be largely investigated. Despite the benefits of cytokines and chemokines produced by TRUCKs for CAR T cells function and endogenous immune system activation [142], it is possible these cytokines and chemokines facilitate melanoma metastasis in the cancer patients. 

Collectively, we discussed the mechanisms involving in melanoma metastasis and further introduced several target antigens that can be targeted via CAR T cells in melanoma (Table 1). Still, it should be noted that many other targets are available in this context, but since they cannot be targeted by CAR T cells, mainly due to lack of surface expression, we did not fully discuss here. For example, various studies have shown that targeting pathways involved in melanoma tumor growth and survival including BRAF and MAPK pathways with specific inhibitors such as vemurafenib and trametinib, respectively, contribute to enhanced overall survival of metastatic melanoma patients [143,144]. Of note, acquired resistance and reoccurrence of disease is the main challenge in this type of therapy [145]. Therefore, we believe that CAR T cells might have an additional advantage compared to conventional inhibitor therapies and could be used in combination therapeutic regimens to enhance overall anti-tumor efficacy in the clinical practice. Moreover, combination therapy using CAR T cells and immune checkpoint inhibitors (e.g., anti-PD-L1) can also be another appealing therapeutic strategy for patients with metastatic melanoma.

## 3. Preclinical and Clinical Studies Using CAR T Cell Therapy in Metastatic Melanoma

In the previous sections we summarized some candidate target antigens in metastatic melanoma and the metastasis process (Table 1). Here we discuss some preclinical studies and clinical trials that have been carried out (or are in progress) on melanoma and metastatic melanoma.

### 3.1. Preclinical Studies

**Figure 2:**
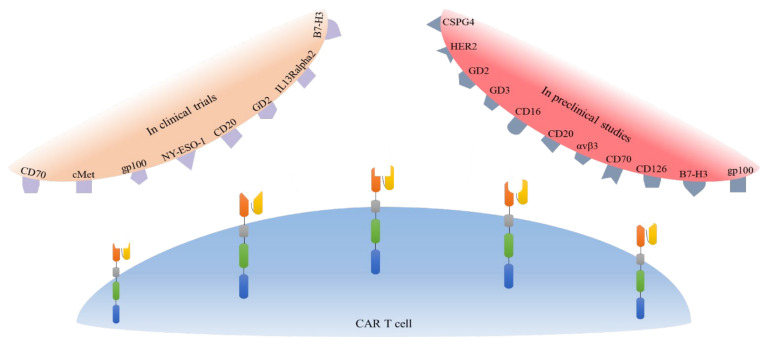
Schematic overview of target antigens for CAR T cells in the pre-clinical and clinical studies. To date, these antigens have been considered as potential targets for CAR T cells in pre-clinical studies and in clinical trials in melanoma patients.

Several pre-clinical studies have laid the foundation for further studies that may progress to clinical trials. CD16, CD126, CD70, B7-H3, HER2, VEGFR-2, gp100/HLA-A2 complex, CSPG4, GD2, and GD3 have been studied as potential candidate antigens for CAR T cells (Figure 2) [147,148,159,161,162,163,164,165,166,167,168,169,170,171,172]. The details are presented in Table 2, and we will discuss some of them in detail below.

CD126 is abnormally expressed in many types of both solid and hematological human tumors. In a dose-dependent manner, CD126-targeted CAR T cells demonstrated potent anti-tumor function in vitro and strong anti-tumor response in metastatic melanoma xenograft mouse model [163].

The chondroitin sulfate proteoglycan 4 (CSPG4) antigen also known as MCSP is strongly expressed on 90% of melanoma cancer cell lines, and other tumors such as glioblastoma, sarcoma, and leukemia. CSPG4 plays a crucial role in melanoma cell proliferation, migration, invasion, and metastasis [173]. A study showed that anti-CSPG4 NKT cells could be transiently transfected using electroporation, with improved activity, and the in-vitro functionality after stimulation with melanoma cells was assessed. Compared to conventional CAR T cells, the anti-CSPG4 NKT cells generated a lower amount of cytokines, such as IFN-γ and TNF, but could still effectively kill melanoma cells [174]. In another study anti-CSPG4 CAR T cells transfected with siPD-1 and siCTLA4 showed reduced expression of PD-1, and were able to secrete higher quantities of cytokines and a good cytolytic effect against the A375M melanoma cell line [174]. In an interesting study, Simon et al. engineered T cells expressing both anti-CSPG4 CAR and anti-gp100 engineered TCR at the same time. These cells generated cytokines and carried out cytolytic activity when encountering their target antigens alone, while no two-sided suppression of the receptors was observed. However, an improved cytolytic effect was observed after co-culturing with target cells that expressed both the target antigens [168].

Inhibition of vascular endothelial growth factor receptor-2 (VEGFR-2) could theoretically inhibit tumor growth by an anti-angiogenic effect [148]. Pre-clinical studies targeting this marker have shown promising results [147,148,149]. The above-mentioned studies have tested different approaches, all with satisfactory results. Chinnasamy et al. utilized CAR T cells targeting VEGFR-2 plus exogenous IL-2. This approach inhibited the melanoma tumor, mediated through attacking the vasculature and not the tumor itself, because the tumor expression of VEGFR-2 was low [147]. Another study combined TCRs that targeted melanoma tumor antigens (gp100, TRP-1, and TRP-2) with VEGFR-2 that targeted the tumor vasculature. They concluded that the simultaneous approach had synergistic effects on tumor eradication, and increased the infiltration and persistence of adoptively transferred tumor-specific T cells within the tumor microenvironment. No significant morbidity or mortality were observed in the mice receiving anti-VEGFR-2 CAR T cells, except those administered with anti-VEGFR-2 CAR T cells and TRP-2 TCR transduced T cells [149]. Inoo et al. utilized CAR-coding messenger RNA (mRNA) delivered by electroporation to produce anti-VEGFR-2 CAR T cells. The mRNA approach resulted in an efficacy of 100% to prepare anti-VEGFR-2 CAR T cells from human or murine CAR T cells for the first few days, without damaging the human T cell activity and phenotypes [148].

A recent study from Yang et al. reported tandem CAR T cells that targeted CD70 and B7-H3, and found that the tandem CAR T cells could simultaneously distinguish two tumor-associated antigens and boost the cytolytic effect against tumor cells, as well as specifically targeting a single antigen. A melanoma mouse model treated by TanCAR T cells exhibited a more pronounced reduction in tumor burden, when compared to controls, and to single CAR (CD70 or B7-H3) groups [164].

The α_v_β_3_ integrin has been found to be over-expressed on a broad range of cancers including, melanoma, breast, prostate, and pancreatic cancer. CAR T cells that targeted αvβ3 were able to eradicate the metastatic A375 melanoma cell line in vitro, and boost the anti-tumor effect in vivo. A single inoculation of anti-α_v_β_3_ CAR T cells in mice was able to inhibit melanoma growth, and increase the long term survival. In summary, anti-αvβ3 CAR T cells killed αvβ3-positive tumor cells rapidly and specifically, secreted IL-2 and IFN-γ, and themselves underwent effective proliferation [152]. 

Overall, the studies discussed above mostly report satisfactory results of various CAR T cell targeting antigens against metastatic melanoma. Another review on this topic also discussed the future promise of several molecular targets for CAR T cell therapy in melanoma, including CSPG4, GD2, CD70, CD20, gp100, and NY-ESO-1 [175]. Nevertheless, achieving this goal requires continuing efforts of researchers to overcome these barriers, and to design the most efficient CAR T cells for future clinical studies.

**Table 2:** Summary of pre-clinical studies involving CAR T cell therapy for melanoma.

Study	Model of Study and Design	Target Tumor Antigen	Findings
[161]	A375 melanoma cell line CD 16 CAR T + CD20 and MCSP antibodies	CD16	Increased cytotoxic activity against target cells
[162]	NOG mice HER2^+^ melanoma cells HER2 CAR T cells	HER2	Her2^+^ melanoma cell killing in vitro and in vivo
[163]	624-mel metastatic melanoma cell line CD126 CAR T cells	CD126	Increased cytotoxic activity against CD126^+^ melanoma cells
[165]	SCID Beige mice HLA-A2-positive Malme-3m cells GPA7-28z-transduced T cells or un-transduced T cells	GP100-HLA-A2 complex	Enhanced cytotoxicity against melanoma cells in vitro Rapid tumor regression, delayed tumor growth and enhanced survival in vivo
[149]	B16 melanoma mice: Group A: Anti-VEGFR-2 CAR T-cells and T cells specific for tumor antigens (gp100, TRP-1, TRP-2)	VEGFR-2	Improved tumor-free survival and enhanced infiltration and persistence of CAR T cells
[148]	C57BL/6 mice B16BL6 cell lines VEGFR-2 CAR-T cells		mRNA Electro-porated CAR-T cells showed similar tumor killing, cytokine production and cytotoxic activity compared to conventional CAR T cells
[159]	NIH-III mice primary and metastatic cutaneous melanoma Tumor cells MCSP or CD20 CAR T cells	CD20 & MCSP	Efficient elimination of melanoma cells co-expressing CD20 and MCSP in vivo
[166]	Rag^−/−^ cγ^−/−^ mice MCSP+ Melur cells CEA CAR T or MCSP CAR T cells	MCSP	Increase survival of mice receiving first & 2nd generation CAR-T cells
[167]	T2.A1; Mel526; A375M cell lines MCSP-specific CAR T cells		Safer activity, similar cytotoxicity and reduced cytokine production of γ/δ engineered T cells against melanoma cells compared to conventional CAR-T cells
[169]	T2. A1 and A375 melanoma cells line CSPG4 (MCSP) specific CAR T cells, gp100 specific TCR α/β T cells or T cells expressing both receptors (TETARs)	gp100 and MCSP	Similar melanoma tumor cell killing capacity, reduced unspecific response and recognition of both antigens by TETARs
[176]	SK-Mel-28 melanoma cell lines anti-GD2 iCAR T cells + Pembrolizumab (PD-1 inhibitor)	GD2	Melanoma cell killing in vitro
[171]	SCID-Luc mice 4405M or P1143 cell lines NT or GD2 CAR T cells		Significant anti-tumor activity of GD2 CAR T cells both in vitro and in vivo
[170]	BALB/c nude mice GD3^+^ M21 cell lines GD3CAR T cells	GD3	Enhanced cytotoxicity, proliferation, and cytokine production of ScFv-CD28/TCRζ receptor expressing T cells
[172]	NSG mice A375-FFLuc cell line B7-H3 CAR T cells	B7-H3	Enhanced survival and significant anti-tumor activity of B7-H3 CAR T cells against Melanoma cells
[164]	NSG mice NCI-H460 or A375 cell lines CD70 CAR2, or B7-H3 CAR T-cells	CD70 & B7-H3	Reduction of tumor burden Increased the overall survival of the mice

### 3.2. Clinical Studies

Owing to the demonstrated success of CAR T cell therapy for melanoma in preclinical studies, researchers are conducting clinical trials to assess the effectiveness and safety of this treatment approach in patients. We identified ten clinical trials involving CAR T cell therapy in patients with melanoma (Table 3). Target tumor antigens included VEGFR2, GD2, cMet, hCD70, gp100, NY-ESO-1, CD20, IL13R-alpha2, B7H3, and bispecific B7H3xCD19 (Figure 2). All of these trials are phase I or II non-randomized, or single-arm trials, which will warrant further investigation if successful.

These clinical trials are novel, and many of them are yet to be completed, with the earliest trial having been started in 2010. Only two studies are completed as of now, and only one of them has available published data. Six studies are still recruiting patients, and two others are suspended or have been terminated.

The only published results come from a single study conducted on 24 patients, with a subset of these patients having metastatic melanoma. VEGFR-2 was the target antigen for the CAR T cells used in this study. Patients received different numbers of CAR T cells in varying numbers of cycles combined with administration of low or high-dose IL-2. The study was terminated because of no observed objective response, and 23/24 patients showing progressive disease. On the other hand, 23/24 patients also suffered from adverse events, with 5 of them being serious adverse events. Three patients had seriously increased alanine transaminase (ALT), aspartate transaminase (AST), and bilirubin, 2/5 had hypoxia, and 1 case of serious pain, infection, nausea, and vomiting occurred [177]. These disappointing results were in contrast with promising results of previous pre-clinical studies that targeted VEGFR-2 in B16 melanoma bearing mice [147,148,149]. One of these animal studies combined anti-VEGFR-2 CAR T cells with exogenous IL-2, similar to the clinical trial [147]. Interestingly, a previous pre-clinical study concluded that co-administration of anti-VEGFR-2 CAR T cells with TCR transduced cells against tumor antigens (gp100, TRP-1, and TRP-2) dramatically improved the tumor-free survival compared to anti-VEGFR-2 CAR T cells alone [149]. Therefore, simultaneous T cell therapies might be more effective in future clinical trials despite the failure of anti-VEGFR-2 CAR T cells in this clinical trial.

GD-2 was the only antigen targeted in two clinical trials [2,7], of which one has been completed, and the results might be published soon. The study added the C7R gene to the CAR T cells to increase their survival and provide a constant cytokine supply [7]. One study tested the anti-GD-2 CAR T cells on blood samples from melanoma patients, and SK-Mel-28 melanoma cell lines [176]. CAR T cells produced significant cancer cell killing, and concurrent PD-1 blockade enhanced their effectiveness. Therefore, we might expect durable responses from these two clinical trials targeting GD2.

Overall, current evidence from clinical studies is very limited in CAR T cell therapy for melanoma and metastatic melanoma, and no CAR-based therapy has yet produced promising results in the clinics. However, the completion of the ongoing clinical trials might decipher the unknowns in this field and pave the path for possible larger clinical trials.

## 4. Challenges and Future Directions

Despite the success of CAR T cells in cancer immunotherapy, several challenges still limit the efficacy of CAR T cell therapy in cancers, including antigen selection and off-target toxicity, antigen loss and heterogeneity, immunosuppressive tumor microenvironment, and insufficient infiltration and penetration of the T cells. Herein, we discuss these challenges and propose some solutions in the context of metastatic melanoma (Table 4).

### 4.1. Antigen Selection and Off-Target Toxicity

Several studies have shown that CAR T cell therapy can result in off-target toxicity in patients. This arises in patients who express the target antigen in their healthy tissues. Off-target toxicity can be reduced by improving the specific recognition of tumor cells and selecting safer target antigens. Melanoma-associated antigen (MAGE) and New York esophageal squamous cell carcinoma (NY-ESO1) have been targeted in metastatic melanoma, as well as in other cancers [182,183,184,185,186]. A few studies have reported neurological toxicity following MAGE-A3 targeting by TCR engineered T cells [187]. Moreover, anti-VEGFR2 CAR T cells were examined in a clinical trial (NCT01218867) with 24 metastatic melanoma patients. The results showed that all patients had disease progression after treatment, except one patient who showed a partial response. Therefore, targeting VEGFR2 seems to be safe in patients, but more research should be performed to improve its effectiveness in the clinic. Another interesting target is CD248. Studies have shown that CD248 is expressed in 86% of metastatic melanoma specimens using tumor microarrays, with no expression in healthy tissues [188]. It is believed that CD248 is involved in the tumor vasculature [189]. Thus, CD248 could be a useful and safe antigen for CAR T cell therapy in metastatic melanoma patients. Despite all the benefits of CAR T cells, safety concerns should be considered, especially in high-dose CAR T cell therapies, and CAR T cell therapies that target the metastasis-associated molecules mentioned in Table 1. Numerous approaches have been proposed by several groups to overcome these problems, including inhibitory CAR T cells [190], dual or multivalent antigen recognition domains, with split signaling [191], and insertion of suicide genes [192]. All these mechanisms could be used to reduce the off target toxicity in CAR T cell therapy.

### 4.2. Antigen Loss and Heterogeneity

One of the least investigated challenges in adoptive T cell therapy is tumor antigen heterogeneity. It is possible that not all the tumor cells express the target antigen, or if they do, the antigen expression is variable. Moreover, immune editing induced by the therapy can further lead to immune escape and tumor outgrowth [193]. Several strategies to overcome this hurdle have been proposed, including the bystander killing effect, using armored CAR T cells, using dual or multivalent CARs, and administration of drugs to up-regulate the target antigens [149,194,195,196,197,198]. Moreover, many studies have reported antigen heterogeneity in metastatic melanoma [199,200], and have discussed the implications for immunotherapy [201]. Still, no antigen loss or heterogeneity has yet been reported in CAR T cell therapy for metastatic melanoma, but this is predicted to occur in the future, after wider clinical use of CAR T cells in metastatic melanoma patients. Other strategies can be used to overcome antigen heterogeneity and loss in CAR T cell therapy, including using epigenetic reversal agents as drugs in combination with CAR T cell therapy. It has been shown that histone deacetylase inhibitors or DNA methyltransferase inhibitors can up-regulate antigen expression in cancer cells. Kailayangiri et al., showed that pharmacological inhibition of Enhancer of Zeste Homolog 2 (EZH2 (EZH2 is responsible for repressive histone methylation in the genome)), induced surface expression of the GD2 antigen in Ewing sarcoma (EwS) cells [202]. They showed that EZH2 inhibition in EwS cells improved anti-GD2 CAR T cell anti-tumor activity. Thus, if antigen loss or heterogeneity was observed to present a problem in the application of CAR T cells in melanoma or metastatic melanoma patients, a combination of epigenetic drugs and CAR T cells could be used to overcome this roadblock.

### 4.3. Melanoma Resistant to CAR T Cell-Mediated Apoptosis

Another obstacle in CAR T cell therapy, especially in the application of CAR T for melanoma patients, is the melanoma cell resistance to apoptosis. This occurs mainly because CAR T cell-mediated tumor cell killing mostly happens through inducing apoptosis in target cells [203]. In line with this notion, it has been established that IGF-1 (insulin-like growth factor 1) plays a key role in inducing resistance to apoptosis in melanoma cells. It has been shown that IGF-1 increases expression of antiapoptotic members of the BCL2 family, and survivin (in both mRNA and protein levels) to protect mitochondria from damage that occurs during apoptosis [204]. Tumor necrosis factor (TNF)-related apoptosis-inducing ligand (TRAIL) mediated apoptosis is another mechanism which immune cells use to kill cancer cells [205]. It has been shown that some melanoma cells in patients are resistant to TRAIL-mediated apoptosis [206]. A recent study has demonstrated that while fully functional anti-CD19 CAR T cells are able to effectively kill tumor cells, the administration of anti-CD19 CAR T cells with a TRAIL inhibitor can suppress the cytotoxic effect of the CAR T cell [207], indicating the importance of TRAIL-mediated target cell killing as a key killing mechanism of CAR T cells. This finding may be applicable to CAR T cell therapy in the context of melanoma; however, it remains to be assessed. Interestingly, it has been reported that apoptosis resistance or partial apoptosis resistance of melanoma cells leads to their increased aggressiveness and metastasis ability by triggering the c-Jun N-terminal kinase (JNK) pathway, as the RNA sequencing signature of JNK pathway activation has been found to be similar to metastatic melanoma. Moreover, it has been demonstrated that partial apoptosis in melanoma cells results in enhanced cell adhesion, chemotaxis, increased melanoma migration and invasion [208]. To overcome this challenge, it is suggested that combination therapy using CAR T cells and (epi)drugs targeting antiapoptotic signaling pathway in melanoma cells could also be an interesting solution. 

### 4.4. Insufficient T Cell Infiltration and Penetration

Another potential obstacle to the clinical efficacy of CAR T cell therapy is insufficient infiltration and penetration of the CAR T cells into the tumor mass. This could be caused by the stromal cells inhibiting the CAR T cell penetration, or interfering with the ability of CAR T cells to infiltrate properly into the tumor bed. Chemokines and their receptors can regulate the infiltration of various immune cells into the tumor site, and have been shown to be involved in tumor progression and angiogenesis [209]. A study looking at metastatic melanoma biopsies showed that up-regulation of several chemokines, including CCL2, CCL3, CCL4, CCL5, CXCL9, and CXCL10 could be correlated with the presence of T cells in the tumor [210]. That study also showed that the corresponding chemokine receptors were also up-regulated in effector T cells. Therefore, up-regulation of chemokines and receptors could be used in CAR T cell therapy in metastatic melanoma patients to overcome insufficient infiltration of CAR T cells into the tumor site. In another study, temozolomide (TMZ), which is the most frequently used drug for metastatic melanoma patients, showed an increase in T cell infiltration in mouse models of transplanted melanoma and genitourinary tumors, but despite a similar increase in CXCL9 and CXCL10 in all sites after TMZ exposure, no increased infiltration of T cells was seen in the cutaneous tumor. This further showed that the combined use of collagenase plus TMZ could induce infiltration of T cells into a cutaneous tumor site [211]. Thus, it can be concluded that stromal cells act as barriers in skin tumors, and the use of CAR T cells that target stromal cells could enhance T cell infiltration into the tumor site leading to a better clinical outcome. Furthermore, it has been shown by Caruana et al., that in vitro expanded T cells lacked expression of the heparinase (HPSE) enzyme. Heparinase can degrade heparan sulphate proteoglycans, an important component of the ECM. They further engineered CAR T cells expressing HPSE. Their results demonstrated improved efficacy of CAR T cells in degrading ECM along with enhanced infiltration and improved anti-tumor activity of CAR T cells [212].

The manipulation of CAR T cells to over-express specific chemokine receptors, and/or using agents to up-regulate chemokine expression in the tumor site, as well as targeting stromal cells by CAR T cells, could result in enhanced infiltration and penetration of CAR T cells into tumors.

### 4.5. Immunosuppressive Tumor Microenvironment

Each cancer has a specific environment in and around itself called the TME. The TME can suppress the cytotoxic activity of immune cells, and thus maintain tumor growth. The TME in metastatic melanoma is complex, with several factors including extracellular matrix, cytokines, growth factors, hypoxic conditions, and various cells such as fibroblasts and immune cells [213,214]. All these factors working together can aid tumor growth and invasion, as well as decrease CAR T cell anti-tumor activity. Several attempts have been made to modify CAR T cells to overcome the hostile TME properties. For example, one study designed PD-1 knock-out TCR engineered T cells specific for the Melan-A antigen. They showed these engineered T cells had higher anti-tumor efficacy, and could delay the progression of PD-L1 positive melanoma tumors in NSG mice [215]. The adenosine produced in the TME, can be used by melanoma cells to evade immune surveillance. Pharmacological or genetic targeting of the adenosine 2A receptor in CAR T cells resulted in enhanced anti-tumor activity of CAR T cells [216,217]. Indeed, targeting other inhibitory receptors in CAR T cells could result in obtaining CAR T cells with superior anti-tumor activity in patients. In another study, the authors engineered anti-VEGFR-2 CAR T cells to constitutively express single-chain IL-12. Their engineered CAR T cells were able to cause regression of established tumors without exogenous IL-2 administration. They showed that anti–VEGFR-2 CAR T cells altered the immunosuppressive TME by reducing both systemic and intra-tumoral CD11b+ Gr1+ myeloid suppressor cell subsets [218]. However, it should be noted that since not all immunosuppressive factors in the TME can be overcome by manipulating CAR T cells themselves, we will discuss additional solutions to the immunosuppressive TME in the “combination therapy” section.

## 5. Combination Therapy

Combination therapies have been investigated, because single therapies in patients showed somewhat unsatisfactory results. As we discussed in this paper, metastatic melanoma cannot be easily treated with a single therapy, even with the best CAR T cells. Therefore, we discuss potential combination therapies that could be used in clinical trials in metastatic melanoma patients to increase their survival. 

One of the most attractive combination therapies is using an oncolytic virus (OV) in combination with CAR T cells. The first and only FDA approved oncolytic virus for the treatment of advanced melanoma is called Talimogene laherparepvec (T-VEC) [219]. Other viral vectors, including Coxsackie viruses, adenovirus, HF-10, echovirus, reovirus, and Newcastle disease virus are currently under investigation [220]. Previous review articles [221,222] have suggested that oncolytic viruses are able to induce secretion of IFN type I in the TME, increase danger signals (PAMPs), reverse tumor immunosuppression, and enhance immune cell infiltration in the tumor site. Use of these viruses could increase CAR T cell infiltration, enhance TAA release by oncolytic virus-dependent tumor cell lysis, and promote the persistence, proliferation, and anti-tumor activity of CAR T cells in solid tumors. Thus, oncolytic viruses might be ideal partners for CAR T cells in tumor eradication. Several studies have pointed out the possible advantages of oncolytic viral therapy in combination with CAR T cells [223,224,225]. However, more investigations in this field should be conducted on melanoma and metastatic melanoma tumors. Guedan et al. used an adenovirus expressing hyaluronidase in a melanoma xenograft model [226]. They showed tumor regression and wide viral distribution following oncolytic adenovirus administration. This concept could be used in combination with CAR T cells to enhance CAR T cell infiltration into skin tumors such as melanoma and prevent melanoma metastasis. 

In a phase I clinical trial using GD2 targeted CAR T cells in metastatic melanoma patients who expressed GD2, up-regulation of LAG-3 and PD-1 expression was observed in stimulated CAR T cells [227]. Thus, using checkpoint blockades in combination with CAR T cells in metastatic melanoma patients might increase overall survival. Another interesting approach to enhance checkpoint blockade efficacy in CAR T cell therapy is the use of armed oncolytic viruses expressing an anti-PD-L1 antibody [223]. This approach could overcome obstacles against CAR T cells in solid tumors, benefiting from both immune checkpoint blockades and oncolytic virus therapy.

A recent study showed that IFN type I secreted in response to oncolytic virus therapy can interfere with CAR T cell anti-tumor activity [228]. They showed that combination use of OVs and CAR T cells injected on the same days did not provide superior anti-tumor effects (compared to either treatment alone) in mice bearing B16EGFRvIII tumor cells. Thus, it may be that precise scheduling of OV and CAR T cell injections could result in achieving better results. 

Immune checkpoint blockade (ICB) is well-established in combination with CAR T cell therapy. Melanoma was the first cancer type to be treated with immune checkpoint inhibitors (ICI) in the clinic. In 2011, ipilimumab (anti CTLA4) gained Food and Drug Administration (FDA) approval for treatment of metastatic melanoma [229]. Shortly after, another ICI pembrolizumab (anti PD-1) was approved by the FDA in 2015 for patients with non-resectable or metastatic melanoma [230]. Nowadays ICIs are considered standard care in metastatic melanoma patients. The combination of nivolumab and ipilimumab was shown to be the most efficient ICI therapy for advanced melanoma [231,232]. However, this combination is associated with high levels of toxicity [233]. In addition, the combination of ICI and CAR T cells has proven to be safe and effective in hematological malignancies [234,235]. Several studies are in progress to assess the safety and efficacy of combinations of CAR T cells and ICI therapy in solid tumors, including glioblastoma (NCT04003649, NCT03726515). Surprisingly, despite the mentioned advantages of ICI and CAR T cell combination therapies, there have yet been no studies or clinical trials to assess the efficacy and safety of combining these two promising anti-cancer therapies in advanced melanoma. Moreover, Chapuis et al., reported a single patient with advanced melanoma whose disease was refractory to both ICI monotherapy (ipilimumab) and monoclonal CTL therapy. However, the combination of IL-21 primed CTLs (MART1-specific CTLs) plus ipilimumab led to a durable complete remission in that patient [236]. Furthermore, clinical trial results have shown that combining antigen-specific CTLs with ipilimumab (CTLA-4 blockade) is safe as well as effective, and could produce durable clinical responses in patients with metastatic melanoma [237]. This result is promising for future combinations of CAR T cell therapies with immune checkpoint inhibitors in advanced/metastatic melanoma patients.

The inhibition or blockade of soluble molecules responsible for promoting metastasis in melanoma, including MMP1, MMP2, and MMP13 is another approach. Another possibility is the over-expression of soluble molecules that inhibit or decrease metastasis in melanoma, including PF4/CXCL4, TR47, and MMP8, which could be used in combination with CAR T cells that target surface molecules.

**Table 4:** Roadblocks and challenges in CAR T cell therapy.

Challenges	Solutions	Reference
Antigen selection and off-tumor toxicity	Using tumor-associated antigens (TAAs) as target antigens in CAR T cell therapy	[183,185,187]
	Control of CAR T cell therapy associated toxicity, including: iCARs, multivalent CARs, and implementation of suicide genes	[190,191,192]
Tumor heterogeneity and antigen loss	Using armored CAR T cells	[198]
	Using bystander killing approach	[197]
	Using drugs, especially epigenetic drugs to up-regulate TAAs	[202]
	Using multivalent CAR T cells	[194]
Insufficient infiltration and penetration	Using CAR T cells that express corresponding chemokine receptors (Suggestion)	[210]
	Using engineered CAR T cells expressing heparinase or other enzymes that can degrade ECM components	[212]
Immunosuppressive tumor microenvironment	Using CAR T cells resistant to immunosuppressive molecules including adenosine	[216,217]
	Using inhibitory receptor knock out/down CAR T cells	[215]

## 6. Conclusions

In this review, we have summarized the mechanisms of melanoma metastasis to discover surface markers that can be used for CAR T cell therapy (Table 1). However, there are still some gaps in our knowledge that need to be fully addressed. So far, plenty of CAR T studies have described the engineering of CARs against cell surface targets, which take part in the metastatic process of melanoma. Some of these engineered CAR T cells have been investigated in preclinical studies and in clinical trials with remarkable results. Moreover, we discussed several potential markers for treatment of metastatic melanoma using CAR T cells alone or in combination with other types of immunotherapy. Nevertheless, more investigation needs to be done in order to evaluate the safety and efficacy of newly proposed targets for CAR T cell therapy. Indeed, in the future a “surfaceome” analysis will need to be done, especially for leukocyte-cancer fusion cells to discover suitable target antigens on these cells that could be used in CAR T cell therapy. There are several challenges ahead involving the selection of target antigens and engineering new CAR T cells. Combination therapies can be a solution to some of these roadblocks. Using CAR T cells in combination with oncolytic viruses seems as if it may be an interesting possibility. Nevertheless, future studies need to be cautious about the possible adverse effects of combination treatments.

## Figures and Tables

**Table 1:** Surface molecules involved in melanoma metastasis, which could be targeted by CAR T cells.

Candidate Targets			Cellular Expression Pattern	CAR T Applicable to All Cancers?	CAR T Preclinical Study in Melanoma	CAR T Clinical Trial in Melanoma
Angiogenic factors		VEGFR1	Endothelial cell	Yes [146]	-	-
		VEGFR2	Endothelial cell	Yes [147]	+ [147,148,149]	NCT01218867
		VEGFR3	Endothelial cell	Yes [150]	-	-
		FGFR1	Endothelial cell	No	-	-
		Neuropilin-1	Endothelial cell	No	-	-
		Neuropilin-2	Endothelial cell	No	-	-
		uPAR	Endothelial cell, Melanoma tumor cells	Yes [151]	-	-
		CXCR1	Endothelial cell	No	-	-
		CXCR2	Endothelial cell	No	-	-
		αvβ5	Endothelial cell	No	-	-
Adhesion molecules	Cadherins	N-cadherin	Melanoma tumor cells	No	-	-
	Integrins	αvβ3	Melanoma tumor cells	Yes [152]	+ [152]	-
		α4β1	Melanoma tumor cells	No	-	-
		α6β4	Melanoma tumor cells	No	-	-
	Ig Superfamily	MCAM/MUC18	Melanoma tumor cells	No	-	-
		ALCAM/CD166	Melanoma tumor cells	Yes [153]	-	-
		NCAM/CD56	Melanoma tumor cells	Yes [154]	-	-
		L1-CAM	Melanoma tumor cells	Yes [155]	-	-
		PECAM-1/CD31	Endothelial cell	No	-	-
MMPs		MT1-MMP/MMP14	Endothelial cell, Melanoma tumor cells	No	-	-
Leukocyte-cancer cell fusions		MET	Endothelial cell, Melanoma tumor cells	Yes [156]	-	NCT03060356
		LAMP1/CD107a	MTFs	No	-	-
		β1,6-branched oligosaccharides	MTFs	No	-	-
		CD163	M2-macrophages, MTFs	No	-	-
		CD204	M2-macrophages, MTFs	No	-	-
		CD206	M2-macrophages, MTFs	No	-	-
		CD44v3	Melanoma stem cells	No	-	-
		MLANA/MART-1	MTFs, Melanoma tumor cells	No	-	-
Embolism		PAR-1	Melanoma tumor cells	No	-	-
Cancer stem cells		CD133	Melanoma stem cells	Yes [157]	-	-
		CD20	Melanoma stem cells	Yes [158]	+ [159]	NCT03893019
		ABCB5	Melanoma stem cells	No	-	-
		CD271	Melanoma stem cells	No	-	-
		ALDH1	Melanoma stem cells	No	-	-
Chemotactic molecules		CXCR3	Melanoma tumor cells	No	-	-
		IGF-1R	Melanoma tumor cells	Yes [160]	-	-
		CXCR4	Melanoma tumor cells	No	-	-
		CCR9	Melanoma tumor cells	No	-	-

Abbreviations: MTFs: melanoma tumor fusion cells.

**Table 3:** Summary of clinical trials evaluating CAR T cell-based treatment for melanoma.

Study	Year of Study	Population and Design	Target Tumor Antigen	Overall Result
NCT01218867 [177]	Started in 2010. Last results update in 2019.	Phase I/II: 24 patients with metastatic cancer, metastatic melanoma, and renal cancer. Cyclophosphamide, Aldesleukin, Fludarabine, and different doses of anti-VEGFR2 CAR T cells (CD8 plus PBL)	VEGFR2	Terminated due to no objective response. 23/24 (95.8%) had progressive and 1/24 (4.1%) had a stable disease. 23/24 (95.8%) had serious or non-serious adverse events. 5/24 (20.8%) had serious adverse events: 3/5 (60%) had increased ALT, AST, and bilirubin; 2/5 (40%) had hypoxia; 1/5 (20%) had pain, infection, nausea, and vomiting.
NCT02107963 [2]	Started in 2014	Phase I: Up to 36 children and young adults with GD2+ solid tumors (melanoma, sarcoma, osteosarcoma, and neuroblastoma). Cyclophosphamide, AP1903, anti-GD2 CAR T cells	GD2	Completed but no published results.
NCT03635632 [7]	Started in 2019	Phase I: 94 patients with several GD2+ malignancies including Uveal Melanoma. Receiving: Cyclophosphamide, Fludarabine, and C7R-GD2 CAR T cells.	GD2	Recruiting patients. C7R gene is added to increase the CAR T cell’s survival and provide a constant cytokine supply.
NCT03060356 [1]	Started in 2016	Early phase 1: 77 patients with malignant melanoma or breast cancer receiving RNA anti-cMET CAR T cell	cMet	Terminated (halt in funding). No results published.
NCT02830724 [178]	Started in 2017	Phase I/II: Adults with five types of CD70 expressing cancers including melanoma. Cyclophosphamide, Fludarabine, Aldesleukin, and anti-hCD70 CAR-transduced PBL.	hCD70	Suspended. No study results published.
NCT03649529 [179]	Started in 2018	Early phase 1: 6 patients with malignant melanoma receiving GPA-TriMAR CAR T cells	gp100	Recruiting patients.
NCT03638206 [5]	Started in 2018	Phase I/II: 73 patients with ten types of cancers including melanoma. Different CAR T/TCR-T regimens, including anti-NY-ESO-1 for melanoma	NY-ESO-1	Recruiting patients.
NCT03893019 [180]	Started in 2019	Early Phase 1: 15 patients with CD20+ unresectable stage III or IV melanoma. anti-CD20 CAR T cells (MB-CART20.1)	CD20	Recruiting patients.
NCT04119024 [6]	Started in 2019	Phase I: 24 patients with stage IIIC and IV Melanoma. Receiving: Cyclophosphamide, Fludarabine Phosphate, Recombinant Interleukin-2, and IL13Ralpha2-specific Hinge-optimized 4-1BB-co-stimulatory CAR/Truncated CD19-expressing Autologous naïve and memory T Cells.	IL13Ralpha2	Recruiting patients.
NCT04483778 [181]	Started in 2020	Phase I: 68 children and young adolescents with several recurrent/refractory malignancies, including melanoma. Receiving: Arm A: second-generation 4-1BBζ B7H3-EGFRt-DHFR CAR T cells Arm B: second-generation 4-1BBζ B7H3-EGFRt-DHFR (selected) and a second-generation 4-1BBζ CD19-Her2tG CAR T cells	A: B7H3 and B: Bispecific B7H3xCD19	Recruiting patients.

Abbreviations: PBL: Peripheral blood lymphocytes, VEGFR2: Vascular Endothelial Growth Factor Receptor-2.

## Abbreviations

ACT: Adoptive T Cell Therapy; AJCC: American Joint Committee on Cancer; ALCAM: Activated Leukocyte Cell Adhesion Molecule; ALDH1: Aldehyde Dehydrogenase; ALT: Alanine transaminase; AST: Aspartate transaminase; bFGF: basic Fibroblast Growth Factor; CAR: Chimeric Antigen Receptor; CRISPR/Cas9: Clustered Regularly Interspaced Short Palindromic Repeats/CRISPR associated protein 9; CSCs: Cancer Stem Cells; CSPG4: Chondroitin Sulfate Proteoglycan 4; CTL: Cytotoxic T lymphocyte; E-Cadherin: Epithelial-Cadherin; ECM: Extracellular Matrix; EMT: Epithelial Mesenchymal Transition; EwS: Ewing sarcoma; EZH2: Enhancer of Zeste Homolog 2; GD2: Ganglioside G2; GD3: Ganglioside G3; gp100: Glycoprotein 100; GPCRs: G-Protein Coupled Receptors; HER2: Human Epidermal growth factor Receptor 2; HGF: Hepatocyte Growth Factor; HLA-A2: Human leukocyte antigen-A2; HPSE: Heparanase; HSCs: Hematopoietic Stem Cells; ICAM1: Intercellular Adhesion Molecule 1; iCAR: inhibitory CAR; ICB: Immune Checkpoint Blockade; ICI: Immune checkpoint inhibitor; IGF-1R: Insulin-like Growth Factor 1; IgSF: Immunoglobulin SuperFamily; ITAM: Immunoreceptor tyrosine-based activation motif JARID1B: Jumonji AT-Rich Interactive Domain 1B; L1-CAM: L1 Cell Adhesion Molecule; LAMP1: Lysosome Associated Membrane Protein-1; LFA-1: Lymphocyte Function Associated Antigen 1; Lu-ECAM: Lung Endothelial Cell Adhesion Molecule-1; Mac-1: Macrophage-1 antigen; MAGE: Melanoma-Associated antigen; MAPK: mitogen-activated protein kinase; MCAM/MUC18: Melanoma Cell Adhesion Molecule; MCSP: Melanoma-associated chondroitin sulfate proteoglycan; MDSCs: Myeloid Derived Suppressor Cells; MITF: Microphthalmia Associated Transcription Factor; MLANA/MART-1: Melanoma Antigen Recognized by T cell 1; MMPs: Matrix Metalloproteinases; MMSC: Malignant Melanoma Stem Cells; MT1-MMP: Membrane type-1 MMP; MTFs: Macrophage-Tumor Fusion cells; N-Cadherin: Neural-Cadherin; NCAM: Neural Cell Adhesion Molecule; NEDD9: Neural precursor cell Expressed Developmentally Down-regulated protein 9; NKT cells: Natural Killer T cells; NSG: NOD Scid Gamma; NY-ESO1: New York Esophageal Squamous cell carcinoma; OV: Oncolytic Virus; PAFR: Platelet Activating Factor Receptor; PAMPs: Pathogen-Associated Molecular Patterns; PAR-1: Protease Activated Thrombin Receptor; PBL: Peripheral Blood Lymphocytes; PD-1: Programmed cell Death protein 1; PDX: Patient-Derived Xenografts; PECAM-1: Platelet Endothelial Cell Adhesion Molecule 1; PF4: Platelet Factor 4; PGF: Placental Growth Factor; PSGL-1: P-Selectin Glycoprotein Ligand-1; ScFv: Single chain variable Fragment; SPARC: Secreted Protein Acidic and Rich in Cysteine; TAA: Tumor Associated Antigen; TanCAR: Tandem CAR; TCR: T Cell Receptor; TETARs: T cells expressing two additional receptors; TILs: Tumor Infiltrating Lymphocytes; TME: Tumor MicroEnvironment; TMZ: Temozolomide; TRP-1&2: Tyrosinase Related Protein-1&2; T-VEC: Talimogene laherparepvec; uPA: urokinase Plasminogen Activator; VCAM-1: Vascular Cell Adhesion Molecule 1; VEGFR-2: Vascular Endothelial Growth Factor Receptor-2; VEGFs: Vascular Endothelial Growth Factors; Vwf: Von willebrand factor.
